# Anemia in Sports: A Narrative Review

**DOI:** 10.3390/life11090987

**Published:** 2021-09-20

**Authors:** Marc-Tudor Damian, Romana Vulturar, Cristian Cezar Login, Laura Damian, Adina Chis, Anca Bojan

**Affiliations:** 1 Faculty of Medicine, Student 5th Year, “Iuliu Hatieganu” University of Medicine and Pharmacy Cluj-Napoca, 8, Victor Babeș Str., 400012 Cluj-Napoca, Romania; marctudordamian@yahoo.com or; 2 Department of Molecular Sciences, “Iuliu Hatieganu” University of Medicine and Pharmacy Cluj-Napoca Romania, 6, Pasteur Str., 400349 Cluj-Napoca, Romania; romanavulturar@gmail.com or adinachis82@gmail.com or; 3 Cognitive Neuroscience Laboratory, University Babes-Bolyai, 30, Fântânele Str., 400294 Cluj-Napoca, Romania; 4 Department of Physiology, “Iuliu Hatieganu” University of Medicine and Pharmacy Cluj-Napoca, 1 Clinicilor Str., 400006 Cluj-Napoca, Romania; 5 Department of Rheumatology, Center for Rare Musculoskeletal Autoimmune and Autoinflammatory Diseases, Emergency Clinical County Hospital Cluj, 2-4 Clinicilor Str., 400006 Cluj-Napoca, Romania; ldamian.reumatologie@gmail.com; 6 CMI Reumatologie Dr. Damian, 6-8 Petru Maior Str., 400002 Cluj-Napoca, Romania; 7 Hematology Department, “Iuliu Hatieganu” University of Medicine and Pharmacy Cluj-Napoca Romania, 400124 Cluj-Napoca, Romania; ancasbojan@yahoo.ca or; 8 Hematology Department, “Prof. Dr. Ioan Chiricuta” Oncological Institute, 400124 Cluj-Napoca, Romania

**Keywords:** sports anemia, iron metabolism, hepcidin, genetic causes of anemia

## Abstract

Recent years have brought about new understandings regarding the pathogenesis of anemia in sports. From hemodilution and redistribution considered to contribute to the so-called “sports anemia” to iron deficiency caused by increased demands, dietary restrictions, decreased absorption, increased losses, hemolysis, and sequestration, to genetic determinants of different types of anemia (some related to sport), the anemia in athletes deserves a careful and multifactorial approach. Dietary factors that reduce iron absorption (e.g., phytate, polyphenols) and that augment iron’s bioavailability (e.g., ascorbic acid) should be considered. Celiac disease, more prevalent in female athletes, may underlie an unexplained iron deficiency anemia. Iron loss during exercise occurs in several ways: sweating, hematuria, gastrointestinal bleeding, inflammation, and intravascular and extravascular hemolysis. From a practical point of view, assessing iron status, especially in the athletes at risk for iron deficiency (females, adolescents, in sports with dietary restrictions, etc.), may improve the iron balance and possibly the performance. Hemoglobin and serum ferritin are measures that are easily employable for the evaluation of patients’ iron status. Cutoff values should probably be further assessed with respect to the sex, age, and type of sport. A healthy gut microbiome influences the iron status. Athletes at risk of iron deficiency should perform non-weight-bearing, low-intensity sports to avoid inducing hemolysis.

## 1. Introduction

Athletes are, by definition, healthy subjects, but they often have out-of-range hematological or biochemical parameters due to physical exercise, training, physiological and psychological stress, environmental conditions, etc. [1]. Certain mechanisms overlap in the pathogenesis of anemia in sports, mostly regarding iron metabolism. Recent years have brought about new understandings with respect to this complex issue. From hemodilution and redistribution considered to contribute to the so-called “sports anemia”, to iron deficiency caused by increased demands, dietary restrictions, decreased absorption, increased losses, hemolysis, and sequestration, to genetic determinants of different types of anemia (some related to sport), the anemia in athletes deserves a careful and multifactorial approach.

## 2. Sports Anemia

Athletes generally have lower hemoglobin concentrations than the general population, called “sports anemia”, a misnomer as it describes false anemia [2]. The decrease in hematocrit (Hct), hemoglobin (Hb), and red blood cell (RBC) count caused by endurance training is explained by an exercise-induced plasma volume expansion, which takes place within few days of intensive training [3,4,5]. In the meantime, the absolute Hb mass is increased as physical effort stimulates erythropoiesis, but this mechanism is outpaced by the plasma expansion [4]. Anemia, defined as a lowered Hb concentration in a venous sample, may be relative or dilutional when the plasma volume is increased, with normal total hemoglobin mass and normal red cell mass [6]. Iron cutoff values for the active population are controversial [7]. Randomized, placebo-controlled oral iron supplementation (100 mg FeSO_4_/day) in iron-depleted female athletes improved the iron status and possibly physical performance [7]. A healthy gut microbiome also influences iron status [8].

A consensus of the Swiss Society of Sports Medicine stated that baseline Hb, Hct, mean cellular volume, mean cellular hemoglobin, and serum ferritin help monitor iron deficiency [6]. In healthy male and female athletes over 15 years, ferritin values < 15 μg indicate empty iron stores, and values between 15 and 30 μg show iron stores are low. In children from 6 to 12 years and in adolescents from 12 to 15 years, the recommended cutoffs are 15 and 20 μg/L, respectively [6]. In adult elite sports, due to increased demands, the cutoff should be 50 μg/L [6]. The tests should be performed at baseline and twice a year [6].

After training, some of the athletes have lower than normal values of hemoglobin, explained by the expansion of the plasma volume in endurance-trained individuals [5]. There are also age-related physiological variations [9]. Adolescent and preadolescent athlete participation in the competition is progressively increasing, and growth spurs along with the effects of the hormonal changes, inflammation, and iron status should be taken into account in this age group [9]. Training can have positive or negative effects on growth, metabolites, enzymes, and hematological variables with respect to the training load, type, and age upon initiation [9]. The hematological parameters may vary over time among athletes and non-athletes [10].

The data regarding the behavior of the hematological parameters are still controversial, depending on the type and length of training [11]. Exercise may result in an acute decrease in hematological parameters other than white blood cells [12]. On the contrary, a study on Brazilian soccer players showed that erythrocyte concentration, Hb and Hct, increased over training time, likely through plasma volume reduction [3]. In soccer players, Hct decreased in 21% of athletes and Hb in 4% during a year of training [13]. In Arab adolescent athletes, generally, the yearly changes in the hematological parameters (Hb, Hct, mean cell volume (MCV), mean corpuscular hemoglobin concentration (MHCH), ferritin) were modest, and the values were higher in the oldest athletes compared to the younger groups [9].

Strenuous exercise causes sustained quantitative changes in blood cell counts and an increment of inflammatory parameters [14] and increases platelet adhesiveness and aggregation, thrombin formation, and activity of coagulation factors [15].

## 3. Iron Deficiency

Iron is an important component of the oxygen-binding proteins, critical in physical performance [16]. Iron deficiency is associated with an alteration of the transport and delivery of oxygen to the tissues, and therefore may affect athletic performance. Iron is also involved in energy metabolism within the electron transport chain, DNA synthesis, oxidative phosphorylation in mitochondria, and ATP production [17,18]. Iron deficiency affects up to 52% of female adolescent athletes [6] and 30–50% of athletes participating in endurance sports [19]. Although the condition is most common in female athletes (15–35%), 5–15% of the male athlete cohorts are also iron-deficient [20]. A high prevalence of exercise-induced iron deficiency anemia can be found mostly in athletes with heavy training loads (e.g., long- and middle-distance runners, rugby players, etc.) [19]. Heavy loads are used during heavy resistance training; explosive type exercise being performed with light loads that are lifted in an explosive manner [21].

### 3.1. Iron Metabolism

**Figure 1:**
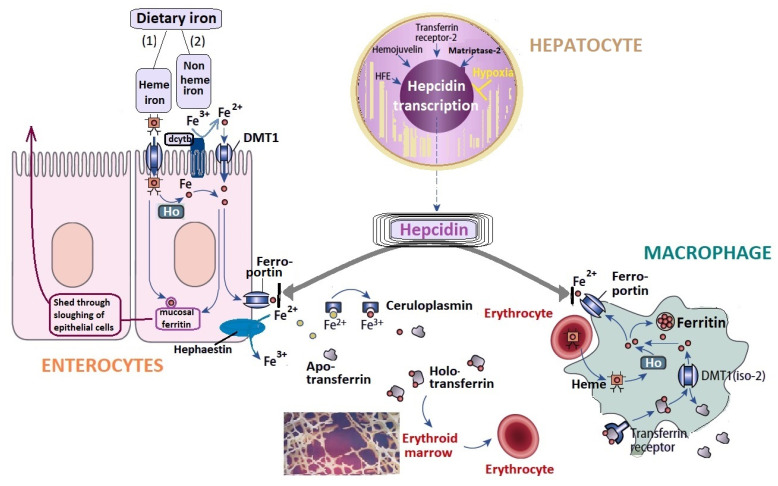
Overview of cellular iron metabolism (adapted from Kowdley et al., 2019, van Hasselt et al., 2016 [23,24]). Legend: DMT1—divalent metal transporter 1; dcytb—ferric reductase duodenal cytochrome B; Ho—Heme oxygenase; HFE—homeostatic iron regulator; HIF—hypoxia induced factors (HIF-1 and HIF-2).

The iron metabolism involves absorption from the duodenal enterocytes, usage in the erythroid precursors, and storage and reutilization in the hepatocytes and tissue macrophages (Figure 1) [19]. Hepcidin is the key regulator of iron homeostasis, as its synthesis is inhibited to facilitate iron efflux in the circulation during increased erythropoiesis [17]. Hepcidin is produced in the liver and degrades the ferroportin transport channel, reducing the ability of macrophages to recycle the iron and thus iron availability [22]. Nevertheless, hepcidin expression is increased by stress and inflammation [17]. Exercise-induced changes in hepcidin and IL-6 are similar in resistance and endurance training [17]. Baseline ferritin and post-exercise IL-6 elevations are key factors in the increase in hepcidin response to exercise [17].

The body thoroughly regulates the absorption, losses, and storage of iron [7,16]. The main mechanisms of iron deficiency in sports are increased iron demand, elevated iron loss, and blockage of iron absorption due to hepcidin bursts [6].

Iron is an essential nutrient in the synthesis of heme (important for hemoglobin and myoglobin structures) and other metalloproteins, such as the iron–sulfur protein cluster, especially important as it plays a crucial role in mitochondrial metabolism; these roles are evidenced by the recent description of several genetic defects in the biosynthesis of iron–sulfur proteins. For all the uses of iron in the organism, a minimum of 20 mg will be required per day, of which only 1–2 mg will originate from intestinal absorption (dietary iron), the rest being re-used. When not bound, iron is toxic; thus, its homeostasis is strictly regulated [23,24].

The primary types of iron in the diet are (1) heme iron, from which it is released through heme oxygenase (Ho), and (2) non-heme iron, which is predominantly ferric iron (Fe^3+^). To facilitate the transport of insoluble ferric iron across the membrane–luminal part of the enterocytes, ferric iron (Fe^3+^) is reduced by the ferric reductase duodenal cytochrome B (dcytb) to ferrous iron (Fe^2+^), which is then transported into the enterocyte by DMT1. The major recycling route for iron is its removal from erythrocyte-derived heme by the enzyme heme oxygenase (Ho), both in macrophages and enterocytes. Once inside the cell, the iron may be stored bound to ferritin or can be exported into the circulation through the transfer across the basolateral part of the enterocytes by the transport protein ferroportin; this protein is responsible for the export of iron into the circulation, both from enterocytes and macrophages. The export process also involves a copper-dependent ferroxidase, hephaestin, which converts ferrous iron back to ferric iron, thus connecting iron and copper absorption [23,24,25,26,27,28]. In the circulation, iron in the ferric state (Fe^3+^) is bound to apo-transferrin, forming holo-transferrin. Both hephaestin and ceruloplasmin influence ferroportin capacity to export ferrous ions into circulation [29]. Hepcidin, synthesized in the hepatocytes. is the key regulator of circulating iron levels, controlling the transfer of iron across the enterocytes and macrophages. Hypoxia is an important regulator of hepcidin metabolism, and the hypoxia-induced factors HIF-1 and HIF-2 inhibit hepcidin activity; these factors are essential in adaptive responses to low oxygen levels, increasing iron bioavailability for erythropoiesis. The main hepcidin stimulatory factors include iron, inflammation/infection, and endoplasmic reticulum/nutrient stress [27]. The synthesis of hepcidin is regulated by proteins, including homeostatic iron regulator (HFE) encoded by *HFE gene*, matriptase-2, hemojuvelin and transferrin receptor 2.

### 3.2. Non Genetic Factors That Influence Iron Metabolism

#### 3.2.1. Iron Absorption

The intestinal *iron absorption* of the iron is influenced mainly by its bioavailability. Iron absorption is diminished in vegetarian diets, and possibly chronic carbohydrates restriction with the purpose of improving performance may also modulate iron metabolism [30]. Dietary iron forms complexes with phytate, oxalate, phosphate, polyphenols, etc., found in high amounts in diets of vegetal origin, thus rendering its absorption more difficult. On the other hand, several other molecules such as ascorbic acid facilitate iron’s absorption. The bioavailability of iron in the diet seems to be more important than the absolute amount of ingested iron. In order to improve iron’s intestinal absorption, it is important to decrease the factors that reduce its absorption (e.g., phytate, polyphenols, etc.) and to increase those factors that augment iron’s bioavailability (e.g., ascorbic acid, etc.) [31,32]. Iron is absorbed in the presence of fermentable carbohydrates that stimulate the growth of bacteria that produce propionic acid and other short-chain fatty acids, thus increasing mineral intake. The studies regarding cereals used as iron fortification foods have shown that flours and derived food products are disadvantageous because of their high phytic acid content, which will decrease iron absorption [33].

#### 3.2.2. Iron Loss during Exercise

*Iron loss during exercise* occurs in several ways: sweating, hematuria, gastrointestinal bleeding, inflammation, and intravascular and extravascular hemolysis [34,35]. Sweating is involved in thermoregulation and is important in physical exercise [36]. Sweating may lead to the loss of up to 2.5 micrograms of iron/L sweat [37]. Hematuria can most likely be encountered in runners who suffered bladder contusions due to the repeated contact of the posterior wall of the bladder with the fixed bladder neck during running [38]. Other mechanisms postulated for hematuria in runners are increased glomerular permeability, renal ischemia, footstrike hemolysis, or a combination thereof [38,39]. Generally, hematuria and proteinuria are transient after exercise, and their causes also include hypoxia, lactate accumulation, oxidative stress, and hormonal changes [39]. Proteinuria and bilirubinuria are potential indicators of acute kidney injury during running [40]. Catecholamines play a role in the hypoxic renal damage and vasoconstriction of the glomerular arteriole, contributing to hematuria [40].

#### 3.2.3. Gastrointestinal Diseases

*Gastrointestinal diseases* in athletes may also influence digestive blood loss. Physical exercise is, to a certain extent, protective against intestinal inflammatory disease; moreover, physical activity may also decrease the disease activity in patients with intestinal inflammatory diseases [41]. Exercise also decreases stress and anxiety related to relapses in this setting [42]. However, in athletes, strenuous exercise may induce intestinal injury, increase permeability and endotoxemia, as well as slow gastric and intestinal motility and malabsorption [43]. The exercise-induced gastrointestinal syndrome results from redistribution of blood flow from the gastrointestinal tract to the working muscles and from the increase in sympathetic activity, which reduces enteric nervous system activity [43]. This syndrome may lead to malabsorption and fecal blood loss and also to alteration of the gut microbiota and systemic inflammatory responses [43]. This could be reversed by maintaining hydration during endurance sports (while avoiding hyperhydration), consumption of carbohydrates according to individual tolerance during exercise, and dietary adaptation of the gastrointestinal tract pre-exercise, including a gluten-free diet in non-celiac individuals [44], avoidance of NSAIDs (nonsteroidal anti-inflammatory drugs), and using several dietary antioxidant supplements [43]. Celiac disease may be a cause of unexplained iron-restricted anemia. Celiac disease is more prevalent in female athletes [45,46] and may be an occult cause of malabsorption contributing to anemia. Moreover, dancers or gymnasts often have traits of hypermobility syndromes, including the Ehlers–Danlos syndrome and others. The Ehlers–Danlos syndrome is an “umbrella term” used for a group of clinically and genetically heterozygous connective tissue disorders, characterized by skin extensibility, joint hypermobility, and variable signs of soft connective tissue fragility [47]. Hematomas or other vascular complications have been reported in the Ehlers–Danlos syndrome, mostly but not exclusively, in the vascular type of the disease [47]. Moreover, the prevalence of rectoceles, hemorrhoids complicated with bleeding, as well as that of diverticular perforation, is increased in the Ehlers–Danlos syndrome [48]. The digestive involvement may overlap with irritable bowel syndrome but may also be the effect of structural abnormalities of the digestive tract such as visceroptosis, hiatus hernia, megacolon, diverticula, or dysautonomia caused by enteric nerve fibers involvement in this setting [49].

#### 3.2.4. Inflammation

*Inflammation* may be involved in sports anemia, as regardless of the exercise type or intensity, IL-6 increases post-exercise [50]. Repetitive bouts of exhaustive exercise induce multi-system inflammation in rats [14]. The increased IL-6 likely triggers hepcidin elevation [50]. Exercise-induced inflammation upregulates hepcidin and consequently lowers the iron absorption in the digestive tract [51]. The exercise-induced hepcidin response in highly trained athletes was not blunted by post-exercise supplementation with proteins, carbohydrates, and vitamins D_3_ and K_2_ in a randomized controlled trial [51]. Hepcidin is increased in patients with inflammatory anemia, as inflammation is a hepcidin activator. Pre-exercise iron status is a master regulator of hepcidin [26,52]. Hypoxia is another regulator of hepcidin, and the hypoxia-induced factors HIF-1 and HIF-2 suppress hepcidin activity and increase iron bioavailability for erythropoiesis [26].

#### 3.2.5. Other Losses

*Other losses*: heavy menstrual bleeding or menstrual symptoms requiring medication to maintain performance are often reported by female athletes [53]. The impact of menstrual cycle phases on athletes’ performance is an important and recently emerged research field related to physical performance [54,55]. Oral contraceptives are also used to control the menstrual cycle and to correct hypermenorrhea [45,56]. Oral contraceptives increase the blood oxidative stress biomarkers and the C reactive protein (CRP) in amateur athlete women [56]. In female athletes, the physiological parameters cannot be simply extrapolated from the high-level athletes according to age and body weight [45]. Oligomenorrhea and amenorrhea range from 3.4 to 70% in sports such as dancing and long-distance running [45,57]. In endurance athletes, amenorrhea is frequent and is associated with a higher cardiovascular training volume [57].

## 4. Sport-Related Hemolytic Anemia

Exercise-induced hemolysis is defined as rupture and destruction of erythrocytes during physical exercise [58]. Intravascular hemolysis during running occurs because of the footstrike, mostly in sports involving running or power walking, due to impact forces [59,60]. In runners, erythrocytes’ lifespan is 40% of that of non-athletes [58]. Bladder contusion also causes hematuria in runners [38]. Hemolysis may cause, mostly in endurance sports, hyperbilirubinemia, even in non-traumatic sports such as endurance swimming, due to muscle contraction and to kidney vasoconstriction, resulting in RBC compression in small vessels [40,58,60]. Proteinuria and bilirubinuria are potential indicators of acute kidney injury during running [40]. Causes for hemolysis are mechanical injury due to forceful ground contacts, repeated muscle contraction, vasoconstriction, and metabolic disturbances (hyperthermia, dehydration, hypoxia, hypotonia, shear stress, lactic acidosis, oxidative damage, proteolysis, increased catecholamines, and lysolecithin) [58]. Moreover, exercise adaptation induces lipid profile changes, including the decrease in free cholesterol and increase in lysolecithin, thus increasing osmotic fragility [58,61]. Other causes, such as pre-existing erythrocytes abnormalities, acidosis, and hyperthermia, may contribute to hemolysis [58]. Haptoglobin and other scavenger proteins clear the low-quantity cell-free hemoglobin derived from exercise-induced hemolysis [58]. Urine dipstick tests may identify the athletes susceptible to acute kidney injury [62]. The reduced hemolysis in low-intensity continuous cycling suggests a protective effect of weight-supported, low-intensity activity against hemolysis [20].

## 5. Genetics, Sport, and Anemia

Alpha-actinin-3 (encoded by *ACTN 3*), a protein belonging to the spectrin family, is a key element in muscle contraction, having structural, metabolic, and signaling functions [63]. It is a sarcomeric scaffold protein that forms a contractile apparatus at the muscle Z line, where it anchors actin filaments together with α-actinin-2 [63]. A polymorphism of *ACTN 3* (R577X, rs1815739) will influence metabolic pathways and muscle phenotype: the XX phenotype is associated with higher metabolic efficiency of the skeletal muscle, but also of the iron metabolism [34]. A marathon race induced in most runners a decrease in RBC, Hb, and Hct, with an increase in hematuria, myoglobin, red cell distribution width, mean corpuscular hemoglobin concentration, mean corpuscular hemoglobin, bilirubin, erythropoietin, and creatinine [34]. Similarly, iron and transferrin levels and transferrin saturation increased immediately after the race and decreased up to 15 days thereafter [34]. A decrease in hematological parameters after an endurance exercise was noted only in RR and RX genotypes of *ACTN3* but not in the XX genotypes [34]. Homozygotes for the 577X alleles form about 20% of the world population and are completely deficient in α-actinin-3 [63]. Interestingly, the frequency of the XX phenotype is higher in endurance athletes [63]. Alpha-actinin-3 deficiency is detrimental for power exercises and sprinting but beneficial for endurance activities [63]. The data are similar in the general population, and the frequency of X alleles is highest in places with low annual temperature, possibly conferring an enhanced cold tolerance advantage or an increased resistance to famine [64].

The *HFE* gene mutations may relate to the increased fitness of an affected individual. For instance, 80% of successful French athletes carry a heterozygous *HFE* mutation (C282Y, H63D, or S65C), suggesting a contribution of the increased iron supply to the performance [65]. Type 1 (or classic hereditary) hemochromatosis is an autosomal recessive disorder characterized by a slow but progressive accumulation of iron in various organs, which becomes clinically apparent during the fourth or fifth decade of life. As many as 0.5% of the Northern European population are homozygous for the C282Y mutation in *HFE*, yet only 5% of male and <1% of female C282Y homozygotes eventually develop liver fibrosis or cirrhosis. Compound heterozygosity for H63D and C282Y of this gene was associated with iron overload [23].

Drug administration that produces chelation, malabsorption, or hemolysis includes tiaprine (an antitumor, iron chelator agent) [66]. There are drugs that can cause drug-induced immune hemolytic anemia (DIIHA): from antimicrobials cephalosporins (ceftriaxone), rifampicin, high-dose therapy with penicillin (>10 days) to anti-inflammatory drugs (diclofenac, mefenamic acid) [67,68,69]. The vast majority of these drugs seem to cause DIIHA only in isolated cases; the incidence was estimated to be ∼ 1 in 1–2 million individuals [69]. 

The beta-thalassemic trait or sickle cell disease affects millions of individuals worldwide and is frequent in some populations and should be taken into account upon the first assessments or during the controls of the athletes with persistent, unexplained anemia [70,71].

## 6. Other Considerations

Diverse strategies for the manipulation of the athlete’s iron status were employed, including those of diet (macronutrients), sex hormones, environmental stress (e.g., hypoxia due to altitude training), types of exercise, and others [20].

Altitude may increase adaptation to hypoxia—used as endurance training in athletes—by increasing RBC number, with the goal to improve performance at sea level [72,73]. An extra iron intake is necessary for adaptation to high altitudes, mostly in winter sports [74,75]. The erythropoietin-induced increases in RBCs or in hemoglobin mass represent adaptive responses to hypoxia [72]. Apart from those mentioned above, other responses induced by hypoxia include angiogenesis, glucose transport and glycolysis changes, pH variations, increased lactic acid tolerance, mitochondrial adaptation, and others [73]. Altitude training increases the iron requirements by 100–200 mg of elemental iron/day [72]. Altitude training can be optimally scheduled during a season in order to improve physical performance [76].

It is a well-known fact that physical effort increases prolactin (PRL) levels as well as other hypothalamic–pituitary–adrenal axis hormones (ACTH and growth hormone GH) [77,78]. In more than half of the athletes, high levels of prolactin are observed [79].

Recent studies on fasting and exercise in healthy men have shown that fasting stimulates the expression of genes involved in iron acquisition and decreases the expression of genes involved with iron storage and export [80]. Intermittent fasting in soccer players might lead to a decrease in the Hb, ferritin, and transferrin levels; though the decrease was statistically significant, the mean values remained within the normal ranges [81].

It is also worth noting that apart from traumatic blood loss with consequences on anemia, hemorrhages in some contact sports, such as boxing, may lead to brain tissue-free iron, triggering iron-mediated oxidative stress and neurodegeneration. To decrease neuronal loss, iron chelation strategies or an increased dietary vitamin E as an antioxidant are being studied to attenuate such long-term consequences [71,82].

High-performing female and male athletes may also be affected by the RED-S syndrome (relative energy deficiency in sports), defined by the International Olimpic Committee in 2014 as a syndrome of health and performance impairment resulting from insufficient caloric intake and/or excessive energy expenditure [83]. The RED-S syndrome was adapted from a previous model, the female athlete triad, characterized by low-energy availability, which negatively impacts reproductive and bone health [78]. This condition may also affect hematologic parameters, immunity, metabolism, protein synthesis, growth and development, endocrine, digestive, cardiovascular, and psychologic functions [83]. RED-S has similarities with Overtraining Syndrome (OTS), both having a hypothalamic–pituitary origin and being influenced by low carbohydrate and energy availability [78]. Low energy availability may be partially induced and may contribute to iron deficiency [84]. Hematological dysfunction, including low ferritin and iron deficiency anemia, were correlated with surrogates for low energy availability in adolescent and young female athletes [84].

## 7. Conclusions

Apart from increased demands, iron reduced absorption, iron sequestration, and losses, as well as other causes of anemia in athletes, are depicted in Table 1.

From a practical point of view, assessing iron status—especially in the categories of athletes at risk for iron deficiency (females, adolescents, in sports with dietary restrictions, etc.)—is important at the beginning of and during the training season. Hemoglobin and serum ferritin are parameters that are easily employable for the evaluation of patients’ iron status. Cutoff values should probably be further assessed with respect to the sex, age, and type of sport. A healthy gut microbiome influences the iron status [8]. Chronic iron supplementation in the presence of normal and high ferritin values is not recommended. Iron supplementation is necessary for altitude training. Athletes at risk of iron deficiency should perform non-weight-bearing, low-intensity sports to reduce supplementary hemolysis.

## Figures and Tables

**Table 1:** Causes of anemia in athletes.

Type of Anemia	Cause	Observations	References
“Sports anemia”	Hemodilution Redistribution (plasma volume expansion)	Controversial; misnomer Increased Hb mass is outpaced by plasma expansion	[2,3,4,5]
Increased iron demands	Increased tissue remodeling	Increased erythropoiesis and muscle hypertrophy	[4,34]
Iron reduced intake	RED S	Low energy availability surrogates correlate with hematological dysfunction	[84]
	Restrictive diets	Dancers, gymnasts, etc.	[85]
Iron reduced absorption	Vegetarian diets	Complexes with phytate, oxalate, phosphate, polyphenols decreasing absorption	[31,32,33]
	Gastrointestinal blood flow redistribution	May result in malabsorption	[43]
	Exercise-induced inflammation	Increased IL-6 triggers hepcidin, consequently lowering the iron absorption	[50,51]
	Celiac disease	Iron malabsorption	[45,46]
Iron sequestration	Inflammation	IL-6 triggered hepcidin contribute to reduced iron availability in acute exercise	[17,43,52]
Iron loss	Sweating	During thermoregulation	[36,37]
	Hematuria	Bladder posterior wall repeatedly kicked against the fixed bladder neck during running, catecholamines, hypoxia, oxidative stress, lactate accumulation, increased glomerular permeability, renal ischemia	[38,39]
	Gastrointestinal bleeding	Decreased gastrointestinal tract blood flow from redistribution to muscles results in fecal blood loss	[41,43]
	Inflammation	Decreased gastrointestinal blow flow leads to intestinal ischemia, increased permeability, endotoxemia, and systemic inflammatory responses	[43,44]
	Trauma	Hematoma, bleeding in contact sports (boxing, etc.)	[82]
	Polymenorrhea	Heavy menstrual bleeding in more than 1/3 of the female athletes Oral contraceptives increase oxidative stress and inflammation	[53,54,55] [45,56]
	Connective tissue fragility	Hematomas, hemorrhoids complicated with bleeding, diverticular perforation, or other vascular complications in hypermobility syndromes (dances, gymnasts, etc.)	[47,48]
	Hemolysis	Footstrike—foot vessels compression and trauma during running Vascular contraction and red blood cells extravascular compression by muscle contraction or by kidney vasoconstriction Lipid profile changes with decreased cholesterol and increased lysolecithin increase osmotic fragility	[58,59] [40,58,60] [58,61]
Genetic causes	*ACTN3* polymorphisms	577X alleles homozygosity results in α-actinin-3 deficiency and improved iron metabolism Detrimental for power exercise and sprinting, but beneficial for endurance activities	[34,63]
	*HFE* polymorphisms	*HFE* mutation (C282Y, H63D, or S65C) increases iron supply to the physical performance	[65]
	Hemoglobinopathies, RBC enzymopathies	β-thalassemia, sickle cell anemia, and others are frequent in certain populations; increased hemolysis	[70,71]

Legend: ACTN3—α-activin-3; HFE—homeostatic iron regulator; RBC—red blood cell.
